# Implications of Veterinary Medicine in the comprehension and stewardship of antimicrobial resistance phenomenon. From the origin till nowadays.

**DOI:** 10.1016/j.vas.2022.100249

**Published:** 2022-03-30

**Authors:** Cristina Vercelli, Graziana Gambino, Michela Amadori, Giovanni Re

**Affiliations:** a Department of Veterinary Science, University of Turin, Largo Paolo Braccini 2, 10095, Grugliasco (Turin), Italy; b Veterinary Practitioner

**Keywords:** Antimicrobial resistance, Companion animals, Food producing animals, Exotic animals, One Health approach

## Abstract

Antimicrobial resistance (AMR) is defined by the entire scientific community as the major threat for human health and it is responsible for an increase in morbidity and mortality rates. The reasons behind this phenomenon are complex and the solution is achievable only considering the One Health approach, that encompasses the integration and implementation of human health, veterinary medicine and environmental status. Authors aimed to write this review to summarize to readers the three milestones of One-Health, underlying the most important topics in which veterinary medicine is mostly involved. Therefore, a short introduction about the history of AMR in veterinary medicine is provided, then more detailed aspects about the impact of AMR related to pets, food producing animals, wild animals and environment are discussed. Finally, some critical aspects about current and future issues are considered.

## Highlights

• Antibiotic resistance is a well-known phenomenon with several implications
• The contribution of Veterinary Medicine is underestimated.
• It was believed that only livestock was responsible for antibiotic resistance.
• Companion animals, wild animals and environment are more involved than estimated.
• Educational tools for public and more veterinary specialists are needed.

## Graphical Abstract

**Figure:**
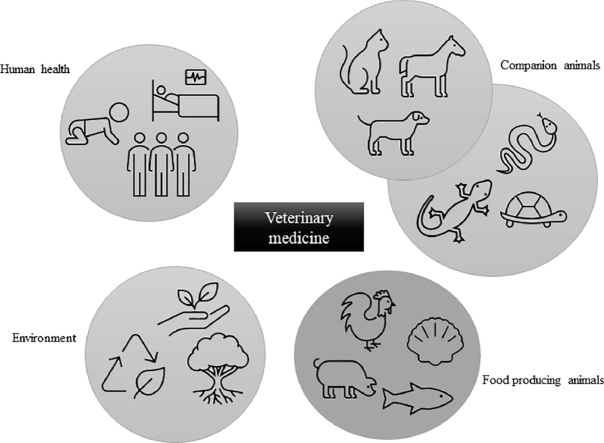

## Introduction

The discovery of penicillin in the 1940s represented a milestone moment to treat people and animals affected by infectious disease. At that moment, few information was available about the mechanisms of action, and none could imagine that bacteria could be resistant to antibiotic drugs. Nowadays it is known that several mechanisms of resistance can occur. Bacteria can be intrinsically resistant due to the absence of specific structure on which antibiotic can act, or could produce enzymes able to inhibit the action of antibiotics or could acquire genes of resistance during evolutionary phase when genetic errors accumulate in the plasmid or chromosome of bacterial cells (called vertical resistance) (Hashempour – Baltork et al., 2019). Horizontal resistance can also occur (called acquired resistance) and encompasses that genetic material can be exchanged within and between bacterial species in which the organisms gain new genes on their mobile genetic elements including plasmids, insertion sequences, phage-related elements and integrons, and transposons (Hashempour – Baltork et al., 2019). The discovery of antibiotic drugs seemed to be a miracle and their massive and uncontrolled use led inevitably to the phenomenon of antimicrobial resistance (AMR) that can be briefly summarized as the capacity of bacteria to survive, even in presence of high concentrations of antimicrobial drugs, and to spread in multiple environments, surfaces and food leading to an increase of morbidity and mortality rates and limited options in drugs’ choice to treat pathologies caused by resistant pathogens (Kumar et al., 2021). This problem is an emergency that requires urgent control measured and is a major concern both for human and veterinary medicine. The current situation is aggravated by the fact that novel antibiotics families will not be released in the next decades (Singh et al., 2017).

The issue is enormous and the forecast about the impact on human lives by the World Health Organization (WHO) is awful: it has been estimated that the deaths caused by antibiotic resistant microorganisms will be 10 million in 2050 (World Health Organization 2014).

The main reasons behind this phenomenon could be linked to the large usage of antimicrobial drugs, lack of awareness among the public and poor public health conditions (Laxminarayan et al., 2016). After the discovery of penicillin, more than 150 antibiotics have been developed and for the large majority of them, a resistance has been reported (Lobanovska and Pilla, 2017). In the last years multi- or pan- resistant strains have been identified and some authors demonstrated that the spreading of these bacteria resistant simultaneously to several antibiotic drugs could be responsible for an increased number of vulnerable people and animals in which even common infections could induce life-threatening conditions (Lobanovska and Pilla, 2017).

In view of the dearth of developments of new antibiotics, several strategies are under investigation to limit the spread of AMR. One of the most important strategies is represented by antimicrobial stewardship, that encompasses to responsibly use antimicrobials, promoting actions that balance both the individual's need for appropriate treatment and the longer-term societal need for sustained access to effective therapy (Dyar et al., 2017). Moreover, other alternative methods are under investigation in order to substitute or implement and potentiate the currently available antimicrobial drugs such as nanoparticles and nanocrystals, bacteriophages, use of sustainable plant and animal origin substances (Kumar et al., 2021). Moreover, new diagnostic and rapid tools are needed for an early identification of pathogens in order to perform a targeted therapy (Lobanovska and Pilla, 2017).

According to the more recent knowledge about the management of antimicrobial resistance, st present, the most efficient strategy is the design and the application of antimicrobial stewardship programs that can vary differently in different settings, influenced by local interpretations but that must be based on the prudent and rational use of antimicrobials, to prevent and avoid overuse, but challenges are still present considering that more detailed surveillance programs, stringent regulatory and direct advocacy of health care professionals is needed (Patel et al., 2020, Dyar et al., 2017).

One Health approach includes a comprehensive and integrative surveillance of microbes in humans, animals, and environment to better understand AMR and develop effective programs to control and prevent this phenomenon (Kahn, 2017). Thus, One Health approach is directed to design and implement programs, policies, legislation and research in multiple sectors to obtain a better public health outcome. The collaboration between different professionals is necessary at local, national, and global levels (Pieri et al., 2020). Veterinary medicine is highly heterogeneous, since veterinarians can work as independent private practitioners (acting alone) or be organized in veterinary clinics or hospitals with multiple staff and they deal with different animal species that include companion animals, food-producing animals and, although less commonly, wild animals (Compri et al., 2020). Veterinarians prescribe antimicrobials to animals as treatment, metaphylaxis, prevention and growth promotion (where allowed and only in certain categories of livestock). In some countries. This specific condition compromises and unbalances all surveillance programs focused on evaluation antimicrobial usage (AMU) (Compri et al., 2020). Even if veterinarians are already involved in One Health approach, in Author's opinion, veterinarians contribution could be enhanced considering skills, competences, and knowledge that sometimes are underestimated.

The present review aims to summarize the origin, the development, and the present situation of AMR, being more focused on the fields of interest of veterinary medicine. Authors are aware about the fact that the topic is enormous and complex. Their aim is to give a specific point of view of a team of veterinary pharmacologists, underlying the importance of the contribution of veterinary medicine in the One Health approach.

## Literature search condition and keywords

**Fig. 1:**
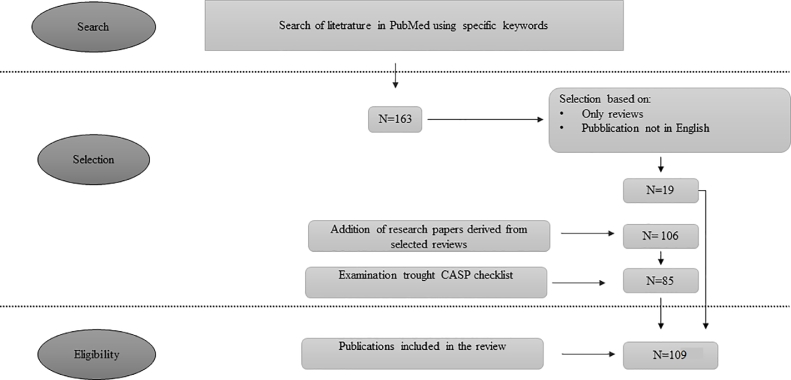
The figure represents the workflow to select literature for the present review.

Relevant literature was systematically selected using the PubMed database. The terms used to search were “antimicrobial resistance”, “veterinary medicine”, “pet”, “dog”, “cat”, “exotic animals”, “dairy cow”, “beef”, “poultry”, “swine”, “food producing animals”, “wild animals”, “milk”, “eggs”, using “AND” as Boolean operator. Eligibility was limited to reviews containing different combinations of the aforementioned words in the title and published in 2020 and 2021. Then, a further selection was performed reading the abstracts and, as the ultimate step, papers cited in results and discussion sections were considered to allow for a critical review. In this last part, also original articles, short communications and case reports, written in English language and published in till December 2021, were enrolled. To be sure that only relevant papers were selected, Critical Appraisal Skill Program (CASP) was applied to each publication. CASP is a checklist that permits critically selecting the literature that can be included in a manuscript, such as systematic review. It is not meant to replace the judgment of the Authors, but it should be intended as a guide. In this specific case, CASP was used to confirm if the selected papers were aligned with the aim of the review. The workflow about literature selection is summarized in Fig. 1.

## From the origin till today

The accidental discovery of penicillin has been conventionally considered as the beginning of the antibiotic era and changed the course of history. Sir Alexander Fleming discovered the antibacterial properties of the mold produced by *Penicillium notatum* in a contaminated petri dish where a culture of *Staphylococcus* was seeded. At the beginning, Sir Fleming speculated that only a local and *in vitro* effect was achievable, as reported by the Author in a paper published in The British Journal of Experimental Pathology in 1929 (Fleming, 1929). Further studies conducted by Drs. Florey, Chain and Heatley at the University of Oxford permitted to evaluate the *in vivo* efficacy of penicillin in mice experimentally infected with group A *Streptococcus* (Lobanovska and Pilla, 2017). This experiment was performed at the beginning of 1940s and provided the key evidence that this was new drug, able to save lives. Nevertheless, even if the high potential was immediately understood, it was hard to find supplies and fundings to perform efficacy and toxicity studies in humans during World War II. The Oxford team was financially supported by the US Department of Agriculture (USDA), permitting the massive manufacturing of the drug that was immediately used to treat and cure thousands of soldiers that have been wounded during the fighting (Lobanovska and Pilla, 2017). The discovery of the molecule of penicillin, the huge efforts to purify it and to make it commercially available to the entire world, were the reasons to confer the Nobel Prize to Fleming, Florey and Chain in 1945 (The Nobel Prize in Physiology or Medicine, 1945). Despite the miraculous discovery, the first demonstrations of antibiotic resistance were early recognized in *Escherichia coli* (*E.coli*) and in penicillin-resistant *Staphylococcus aureus*, that are strains able to produce penicillinase, an enzyme able to destroy the beta lactam ring of natural penicillin. The introduction of the semisynthetic methicillin blocked for a while the resistance phenomenon that restarted very early, and it was evident in *Staphylococcus aureus* methicillin resistant strains. Between 1961 and the end of 1990s, several mechanisms of resistance have been discovered and reported for *Pneumococcus, Gonococcus* and *Enterobacteriaceae* strains (Hartman and Tomasz, 1981; Lowry, 2003; Nordmann, 1998).

In the same years of the discovery of penicillin, sulfanilamide, a sulfonamide derivative, was used to treat bovine mastitis, considering specifically its pharmacodynamic properties (Roach and Hignett, 1945). From that moment on, several sulfonamides have been discovered and distributed all over the world. Another step forwards the development of new antibiotic drugs was the combination between sulfonamide and diaminopyrimidine that permitted an optimal synergy to treat several pathologies. Currently, in veterinary medicine, sulfonamides and their combinations are still used as first line therapy: they are listed among class D compounds of Antimicrobial Advice Ad Hoc Expert Group (AMEG) classification. This is a low-class risk for public health and should be taken into consideration as first choice to treat animals.

The consciousness about the risks related to antimicrobial usage arose in late 1960s, when the Swann report was published and, according to the obtained results, it was proposed to ban the administration of penicillin and tetracycline as growth promoters. It was a milestone moment that was considered for the development of the current European regulatory system. Similar considerations were made by the task force on antibiotics of the Food and Drug Administration (FDA) about the possible danger for consumers related to AMR derived from food producing animals. Nevertheless, at that time the feed industry objected that this kind of evaluations were unfounded conjectures. This opposition was politically strong and permitted to ignore the problem for several years (Lees, Pelligand, Giraud, & Toutain, 2019).

Overall, the comprehension about the mechanisms responsible for the acquisition of resistance was not always clear and straightforward. In the middle of 1990s, the concerns about the possible transmission of AMR from animals to humans were focused on the use of avoparcin in food producing animals. This drug is a glycopeptide, related to vancomycin and teicoplanin (that are essential drugs in human medicine to cure serious gram-positive bacterial infections), was used as a growth promoter in poultry, pigs, and cattle in Europe, but it was forbidden in the USA. An increasing prevalence of infections caused by vancomycin-resistant enterococci (VRE) in humans was noted in Europe and it was claimed that the use avoparcin in livestock was the main responsible for the passage of resistant bacteria from animals to humans. This pushed authorities to ban avoparcin in food producing animals. Curiously, in the same years, in the USA the risk for people to be infected by VRE was very high but this was explained by the over-consumption of glycopeptides in hospitalized patients. The opposite situations in Europe and USA did not find a unique solution: no robust evidence was found to support the hypothesis that the spread of VRE was related to the consumption of avoparcin in livestock but, in order to protect the consumers, the ban was applied in Europe (Acar et al., 2000; Lees, Pelligand, Giraud, & Toutain, 2019).

In other cases, the passage of antibiotic resistant bacteria between food animals and humans was supported by strong evidence and was easier to understand. Between 1980s and 1990s, nourseothricin, an aminoglycoside, was used as growth promoter agent in pigs in Germany. After a few years, it was identified a plasmid borne resistance in *E. coli* from nourseothricin fed pigs that was disseminated in the environment through manure and contaminated river water. The same resistant bacteria were isolated in the gastrointestinal tract of farmers, farm employers and their family members. The resistant determinants were detected in *Shigella* and *Salmonella* strains isolated in human diarrhea cases and, since Shigella is a pathogen of primates, it was deducted that an horizontal gene transfer responsible for aminoglycoside resistance occurred in the bowel of humans (Kirchhelle, 2018). Another emblematic case which was useful to demonstrate the close correlation between antimicrobial drug use and the occurrence of AMR in humans was that of the ceftiofur resistance *Salmonella enterica* serovar Heidelberg in Canada at the beginning of the new century. It was allowed to administer this antibiotic drug directly in hatcheries and, in the periods when ceftiofur was used, a concomitant increasing prevalence of human infections caused by *Salmonella enterica* ceftiofur-resistant was identified. The first hypothesis about the possible cause was focused on the use of this cephalosporin in hatchery and it was decided to apply a withdrawal period that corresponded to a decrease of human *Salmonella* infections. The confirmation was found when the administration was reintroduced and the human infections increased again (Lees, Pelligand, Giraud, & Toutain, 2019).

In the European Community, concerns about the possible passage of AMR bacteria or resistance-genes from animals to human, led to a wide ban in 2006 of all animal feed additives containing antibiotics and limited or forbid the use of antibiotic drugs for non-therapeutic purposes, i.e., as growth promoters (Regulation EU 2003/1831). It was expected to reduce the therapeutic failures in human and veterinary medicine and to lower the incidence of foodborne diseases in humans: these goals were partially reached and, unfortunately, it was not possible to demonstrate a compensatory increase in therapeutic efficacy of antimicrobial drugs in humans. European community was the first to apply a strict regulation about the non-therapeutic use of antibiotic drugs in food producing animals, followed by the ban of FDA in 2017 that forbid the use of antibiotics as feed supplements, and followed by China, that decided to forbid the use of antibiotic drugs as growth promoters in livestock from 2020 (Lees, Pelligand, Giraud, & Toutain, 2019).

## Antimicrobial resistance in humans

The professional oath for Veterinarians in Italy enunciates:

*[...]prometto solennemente di dedicare le mie competenze e le mie capacità alla protezione della salute dell'uomo, alla cura e al benessere degli animali [...], di promuovere la salute pubblica e la tutela dell'ambiente [...]secondo scienza e coscienza, [...].* that can be translated as:

*[...]I solemnly swear to dedicate my skills and abilities to the protection of human health, to the care and well-being of animals, [...] to promote public health and environmental protection; [...] according to science and conscience, [...].*

Authors have chosen to write part of the professional oath of Italian veterinarians to offer food for thought to readers on how central human health and public health are for veterinarians. The veterinary setting is complex and includes interaction with the human sector, even in the antibiotic drug use, the surveillance program, and the activities to limit AMR, that are mainly resumed in One Health Approach. Authors thought that it is important to give to readers a short explanation about AMR in humans and to circumscribe the role of veterinary medicine in relation to human health.

As mentioned along the introduction, the discovery of penicillin in the 1940s and the usage of antimicrobials were considered a miracle to save people's lives. At first there was no perception about AMR and its consequences that were perceived belatedly (Pieri et al., 2020). Public expected a lot from this kind of drugs and physicians were pushed to prescribe antibiotics in an inappropriate way even for non-infectious diseases (Pieri et al., 2020).

Methicillin- resistant *Staphylococcus aureus* (MRSA), VRE, extended spectrum beta lactamase (ESBL) and high level *Enterobacteriaceae* producing AmpC enzymes and carbapenemase produced by *Enterobacteriaceae, Pseudomonas aeruginosa* and *Acinetobacter baumannii* producing carbapenemase are pathogens that have acquired specific and worrying resistance, that nowadays represent the main public health concerns (Santajit and Indrawattana, 2016; ECDC, 2021). The aforementioned phenotypes are frequently associated with multi drug resistance (MDR) bacteria, that means that the bacteria are resistant to more than one antibiotic class. This can be explained by the fact that resistance genes are located on mobile genetic elements that are able to move within or between DNA molecules, like transposons, gene cassette, integrons, plasmids and integrative conjugative elements (Partridge et al., 2018). These mechanisms are not exclusive for human pathogens and can be transferred also among zoonotic bacteria. In case of horizontal transfer, the resistance genes are passed to neighboring bacteria and the passage of resistance can be greater, such as in *Enterobacteriaceae* and Gram-negative pathogens (Carroll et al., 2019).

The attention of several investigations has been focused to find a correlation between antibiotic consumption in animals and the acquisition of resistance in pathogenic bacteria responsible for serious infections in humans. Nevertheless, an important part of the worst infections is identified in hospitals and healthcare facilities, high-risk points for patients and health professionals, due to the fact that several MDR pathogens can be hosted and are responsible for community-acquired-infections, leading to an increase in morbidity and mortality of patients, increase of healthcare costs and, controversially, a major use of antimicrobials drugs (van Duin and Paterson, 2016). On the other hand, the main non-human sources of antimicrobial resistance include the manipulation of pets, large animals and wild animals, lack of access to clean water, poor hygiene measures, eating contaminated food or being exposed to a contaminated environment (for example, sea water). In order to better clarify all these arguments, the following paragraphs will go deeper into each topic.

## Antimicrobial resistance in food producing animals

The widespread of antimicrobial resistance in food-producing animals includes the possibility to share and diffuse microorganisms able to cause disease in humans. The phenomenon of AMR complicates the current situation of food-borne disease: it was estimated that every year, 600 million infections and 420,000 deaths are caused by foodborne pathogens, mostly children (WHO, 2015). Due to AMR, the therapeutic options are limited and the risk to increase the morbidity and mortality rates of foodborne diseases is concrete (Hashempour – Baltork et al., 2019).

Considering the first uses in history of antimicrobial drugs, it is not possible to distinguish the use of antibiotic drugs in agriculture or in husbandry. This was due to the fact that antibiotic drugs were managed by agronomists and not under the direct control of veterinarians or veterinary pharmacologists, with an incorrect usage of these drugs that were mainly administered as growth promoters or to prevent disease (Lees, Pelligand, Giraud, & Toutain, 2019). It was believed that the massive use was necessary to increase meat production, that quadrupled in the past 50 years. Antibiotic drugs have been administered for long periods to improve feed conversion or to prevent disease: these non-therapeutic uses permit an incredible selective pressure on bacteria that acquired new strategies to survive, that are the mechanisms responsible for antibiotic resistance (Kirchhelle, 2018). The irrational use of antimicrobial drugs continued and in 1951, FDA allowed the use of antibiotics in animal feed without a veterinary prescription (Lees, Pelligand, Giraud, & Toutain, 2019). According to the modern scientific knowledge, this decision seems to be wired but contextualized after the end of World War II, it aimed to improve the production of animal protein using inexpensive techniques, as it is nowadays mirrored in low-income countries (Hao et al., 2014; Kirchhelle, 2018). Despite this lesson from the past, it has been forecasted that countries such as Brazil, Russia, India, and China will increase in a significant way their meat production. In order to achieve such a great goal and maintain the prices affordable, it was predicted that antibiotic consumption of these countries will increase significantly (Van Boeckel et al., 2015). Moreover, it is not possible to separate the administration of antimicrobial drugs in livestock from the use in agriculture: the two systems have been and still are closely related. Antimicrobial drugs are used to implement vegetable and fruit production, to increase the production of cereals and fodder, and they can be added to preserve food and extend the storage period (Kirchhelle, 2018).

According to the WHO (WHO, 2016), antibiotics can be ranked in different categories for food producing animals:

1 Therapeutic: antibiotics that can be used to treat animals with clinically diagnosis of infectious disease or illness.
2 Disease prevention: antibiotics administered in healthy animals considered to be at risk of infection or prior to the onset of symptoms correlated to an infectious disease. This includes both prophylaxis and metaphylaxis. This is a common situation in transportation of young animals (i.e., beef) or animals bred in crowded farms (i.e., swine).
3 Growth promotion: antibiotics that are administered at sub therapeutic concentrations to increase the rate of weight and the efficiency of feed conversion. The mechanisms responsible for this effect have not been clearly identified. Some theories proposed that antibiotics could alter gut microbiota reducing the competition for nutrients, between host and commensal bacteria improving nutrient absorption and reducing the number of pathogenic bacteria (Giguère et al., 2013).

The continuous improvement of scientific knowledge led to the awareness that it is mandatory to reduce the use of antimicrobial drugs. This is achievable thanks to a rational use and through a careful application of antimicrobial stewardship programs (ASPs). Considering as a milestone concept the decrease of resistance in animals and humans, without reducing farm productivity (Patel et al., 2020). In Europe, several countries decided to ban the use of antibiotics for disease prevention, improving surveillance about antibiotic utilization, and setting national reduction targets or implementing the prescription methods using computerized protocols (Patel et al., 2020; Vercelli et al., 2021). An example about the application of ASP is represented by Denmark, that since 1996 has been reporting antibiotic usage and resistance in humans and livestock. Another example is Belgium, that was able to reduce antibiotic usage up to 50% in the last 10 years (Jensen et al., 2014; More, 2020). In the United States, antibiotics are no longer permitted as growth promoters but can be prescribed in an easier way, without a strict control, as normally stated in Europe: it was supposed that two thirds of the tonnage of antibiotics considered medically important for humans are sold and used in food producing animals and these factors contribute to the increasing of antibiotic resistant infections in humans (Patel et al., 2020).

A major point of contention between WHO and USDA is that the latter never recognized the need to cease the use of antibiotics to prevent diseases in livestock. Taking advantage of this situation, some companies just re-labeled their products containing antibiotics and claimed these products only for their preventive features: this permitted repurpose these products on the market but these drugs were still administered to induce as growth promotion, hiding behind a legal quibble US Dept of Agriculture (USDA) 2021. Moreover, it is also allowed for farmers to administer antibiotics to animals, following the guidelines of the veterinarian but not under a direct control: this underlines the shortcomings of the US surveillance program (Patel et al., 2020). In this condition, a strong request to implement regulation is performed by consumers and associations that are encouraging restaurants and groceries to choose meat obtained from animals raised without antibiotics and are also asking for a more detailed and clearer label (Patel et al., 2020).

The European Union already banned the use of antibiotics as growth promoting agent in 2006 through the application of Regulation 2003/1831, and the new regulation adopted in January 2022 (Regulation EU 2019/6) includes a ban on the preventive use of antibiotics in animals, extended also to medicated feeds, denying metaphylaxis and establishing the obligation of a careful surveillance collecting data about sales and consumption in order to preserve antibiotics for humans in all countries belonging to European Community (EC Reg. 6/2019). According to the classification performed by WHO (Table 1), antibiotics encompassing in the highest - priority critically important antimicrobials (CIAs) for human medicine should not be used in food producing animals. Moreover, new classes of antibiotics that will be discovered to treat humans will be considered as critically important and their usage will not be allowed in livestock (WHO, 2016). Bovine and swine species have been commonly understood as food producing animals but other species such as broilers, turkeys and fish are not to be excluded, considering their economic impact and their worldwide distribution as a cheap source of animal proteins.

**Table 1:** The table compares the main antibiotic families categorized by Antimicrobial Advice Ad Hoc Expert Group (AMEG) and World Health Organization (WHO). Following the AMEG classification, the categories are A= avoid, B= restrict, C=caution and D= prudence. The parallel Who ranking from the highest to the lowest degree is critical important antibiotics highest priority, critically important antibiotics high priority, highly important antimicrobials, important antimicrobials.

	AMEG	WHO
Aminoglycosides	C	High Priority
Cephalosporins 3°-4°	B	Highest Priority
Macrolides	C	Highest Priority
Penicillins	D	Highly important
Polymyxin	B	Highest Priority
Quinolones	B	Highest Priority
Tetracyclines	D	High Priority
Amphenicols	C	Highly important
Aminopenicillins	C	High Priority
Rifamycins	C	High Priority
Cephalosporins 1°-2°	C	Highly important
Lincosamides	C	Highly important
Pleuromotilins	C	Important
Streptogramins	A	Highly important
Sulfonamides	D	Highly important
Glycopeptides	A	Highest Priority
Oxazolidines	A	High Priority

The potential risk correlated to the transmission of resistance genes or resistant bacteria with food, is also linked to aquaculture. Mussels contaminated with Gram negative carriers of ESBL or *Klebsiella pneumoniae* producing carbapenemase (KPC) have been identified in North Africa markets, and it was reported the presence of *mcr-1* genes in *E.coli* in Norway. The increasing request of raw fish for sushi and sashimi in Europe, highlighted the necessity for strict controls in food safety (Mani et al., 2017; Silva et al., 2019). It has been described that, in 2014, China produced over 45 million metric tons of fish, crustaceans, and mollusks and about a half of this quantity has been exported. The massive use of colistin in Chinese aquaculture has generated plasmid-mediated colistin resistance genes *mcr-1* and *mcr-2* in *Aeromonas, Shewanella* and *E. coli* that can be transmitted to humans through the food chain (Cabello et al., 2017; Pieri et al., 2020). Another report highlighted the extremely high prevalence of *Bacillus cereus* resistant to rifampin and to most beta lactams isolated in aquatic products (Zhang et al., 2020).

Poultry is one of the most widespread types of meat consumed worldwide and antimicrobial drugs have been extensively used to prevent diseases and as growth promoters (Nhung et al., 2017). The increasing concern in AMR in poultry, especially against fluoroquinolones, is worrying not only for the treatment failure and economic losses but also because of the possibility to spread zoonosis: poultry is considered to be the main host of *Campylobacter* that can cause acute bacterial enteritis in human beings. Fluoroquinolones in poultry have been used without a criterion in the 1990s in Australia and this led to an increasing rate of resistance in *Campylobacter* (Lees, Pelligand, Giraud, & Toutain, 2019). This was not seen with the same gravity in other countries (i.e. Europe) where a stricter regulation was applied (Cheng et al., 2012). Other interesting and concerning results have been described about the possible extra chromosomal resistance against colistin that has been identified in broilers in Italy. Colistin is a last resort drug and a resistance to this antibiotic implies that few therapeutic options could be available for invasive infections in humans caused by ESBL and Salmonella that can be transmitted through the food chain (Carfora et al., 2018)

Handling and consumption of contaminated chicken meat have been described as the common modes of transmission of AMR bacteria leading to infections in humans (El-Hack et al., 2021). The increasing emergence of AMR also in these food producing animals has led to finding alternative strategies to limit this phenomenon. The most important strategy is represented by antibiotic stewardship program, specifically addressed to this situation and that include better biosecurity measures, distribution of drinking water with antimicrobial properties, administration of bacteriophages, application of vaccination protocols and also better hygiene measures during slaughtering (El-Hack et al., 2021). These procedures are fundamental also considering the fact that it was described that plasmid mediated by ESBL/pAmpC-producing bacteria can be transmitted in broilers vertically, horizontally, in hatchery and among farms and the acquisition of resistance is extremely fast also in commensal *E.coli* (Dame-Korevaar et al., 2019; Lee et al., 2017).

Concerns about AMR are related not only to meat consumption but also to derivates of animal origin such as milk and eggs.

Antibiotics are frequently used to treat mastitis in dairy cows. Aminoglycosides and beta lactams are the most commonly used molecules and can remain undegraded in milk or can be dispersed in the environment (Pietschmann et al., 2020). Consumption of contaminated milk could be responsible for development of AMR or hypersensitive reactions in human beings (Blumenthal et al., 2019; van Duijkeren et al., 2019).

Recently, insects have been enrolled as edible products, as alternative source of energy and the high-quality protein content. It has been demonstrated that cockroaches, houseflies, ants, and mosquitoes can harbor AMR (Gwenzi et al., 2021). The same worrying phenomenon has been recognized for wild insects that can share habitats with humans and can carry on AMR from environment to humans (Gwenzi et al., 2021).

## Antimicrobial resistance in pets

Antibiotics are very often used in the clinical practice of companion animals, considering that the number of pets has been growing substantially over the last decades and people asks for the same level of care and cure expected for a family member (Guardabassi, 2004; Singleton et al., 2017; Singleton et al., 2020). They represent a crucial point in the transmission of AMR through direct contact, bites, scratches, and licks and considering that they share lifestyles, habits, and spaces with their owners (Bandyopadhyay and Samanta, 2020; Gwenzi et al., 2021; Pomba et al., 2017; Singleton et al., 2020). As previously established for other sources, also the indiscriminate use or over-usage of antibiotics over the past years has greatly enhanced the AMR, resulting in strong selective pressure with reduced sensitivity or acquisition of resistance to several antimicrobial families at the same time (Aworh et al., 2019; Damborg et al., 2016). This can compromise the therapeutic success in companion animals, waste of money and time for owners and an increase of mortality rate (Aworh et al., 2019; Lebreton et al., 2014). The most worrying resistant bacteria shared between pets and humans are methicillin-resistant *Staphylococcus pseudintermedius*, methicillin-resistant *Staphylococcus aureus, Enterococcus faecium* and *faecalis,* and ESBL (Pomba et al., 2017; Prestinaci et al., 2015).

Pets and owners have long life expectancy, have similar pathologies, and can undergo to similar therapeutic protocols using the same classes of antibiotics: this led to some important considerations about the fact that in small animal practice, CIAs can be used representing a major threat of AMR for humans (World Health Organization 2019; Collignon and McEwen, 2019; Tompson et al., 2021). The decision to use antibiotics in pet clinical practice is linked to the treatment of a single sick patient and less frequently for prophylactic purposes (Collignon and McEwen, 2019). According to the guidelines, antibiotics should not be prescribed for clean surgeries carried out in asepsis. Nevertheless it has been described that sometimes veterinarians prefer to administer amoxicillin alone or in combination with clavulanic acid to prevent potential infections in the postoperative period (Mateus et al., 2014). In order to use antibiotics in a prudent and correct way, veterinarians should limit their use only to infections sustained or complicated by bacteria, choosing from those registered for the target species and pathology (Mateus et al., 2014; Singleton et al., 2017). In the event that there is no availability of a particular antibiotic, the veterinarian can prescribe an off-label antibiotic in exemption according to the cascade rule, including antimicrobial drugs for human use (Papich, 2021; Singleton et al., 2017). Often in clinical practice due to the need to initiate therapy, the veterinarian may decide to set up empirical therapy, considering that the optimal choice is an antibiotic with a narrow spectrum of action (Joint scientific report of ECDC, EFSA and EMEA, 2009; Singleton et al., 2020). However, this should be limited as much as possible by a correct diagnosis and the guidelines that assist the veterinarian choosing the most appropriate antibiotic to use, but a complete uniformity in protocols, and in guidelines are not defined but will be with the new European regulatory (Papich, 2021; Regulation EU 2019/6; Singleton et al., 2020; Tompson et al., 2021). In all cases, it is important to prescribe an antimicrobial agent considering the clinical signs shown by the patient, pharmacological criteria such as the pharmacokinetic and pharmacodynamic characteristics of antibiotics and microbiological criteria (Rodríguez-Gascón et al., 2021).

Data obtained from a recent British study by Singleton and colleagues, according to the Australian investigation conducted by Hur and his team, showed that in cats the most commonly used molecule is cefovecin, due to its broad-spectrum and its long-acting effect after a single injection, despite belonging to the third-generation cephalosporins that are considered of highest priority in the CIAs group (HPCIAs) (Hur et al., 2020; Singleton et al., 2020; World Health Organization 2019). In contrast to the feline species, the combination of amoxicillin and clavulanic acid is the most widely used antibiotic in the canine population, even though this antibiotic belongs to the class C of AMEG classification, and it should not be used as first choice drug according to the guidelines (European Medicine Agency (EMA)/688114/2020 2020; Hur et al., 2020; Singleton et al., 2020).

Veterinarians play a key role in the correct management of antibiotics and education of people, considering the lack of awareness among the pets’ owners about antimicrobial agents and AMR (Middlemiss, 2018). Owners often request the prescription for these medicines believing that this is the correct way to care for their animals, sometimes erroneously thinking that not giving an antibiotic is not treating their animals, whereby it is crucial that vets establish a trustful relationship and a good communication with their clients (Middlemiss, 2018; Smith et al., 2018). It is essential for pet owners to realize that antibiotics have only to be administered if strictly necessary, in order to avoid counterproductive effects linked to the spread of antimicrobial resistance on their own and their animal health (Smith et al., 2018).

A deeper understanding of the phenomenon of AMR would ensure a greater focus on another critical point concerning errors in antibiotic intake (Prestinaci et al., 2015). Mistakes such as discontinuing treatment when clinical symptoms disappear and dosages not respected are made by humans when performing medical antibiotic therapy for themselves and when they have to treat their animals (Prestinaci et al., 2015; Smith et al., 2018). Owners can contribute to their pet's wellbeing by carrying out preventive vaccination and antiparasitic measures, as a healthy animal that is checked regularly is less likely to develop infections (Singleton et al., 2020). Nevertheless, they are responsible for the administration of antibiotic treatments to their animals, and they should be targeted in educational activities in order to foster appropriate use of antimicrobials in accordance with veterinarian prescription (Compri et al., 2020). In the past years there was a misconception that the AMR problem was largely related to the use of antibiotics in the livestock of food-producing animals, while today companion animals are also recognized as responsible for the spread of this phenomenon (Pomba et al., 2017). This sectoral approach is also found in legislation, referring to European Commission Decision 2013/652/EU dedicated to assessing the risk of transmission of commensal and pathogenic bacteria from food producing animals to humans, whereas there is no corresponding law for pets (Timofte et al., 2021). Also in this case, One-health approach is the only way to combat the serious global threat of AMR, through better management of weak points such as the need for globally shared and standardized legislation between all the countries to balance antibiotic use and monitoring antibiotic resistance (Gwenzi et al., 2021; Prestinaci et al., 2015). Furthermore, cross collaboration is needed between physicians and veterinarians, who are responsible for prescription of antibiotics, chemists who dispense the drug and customers, and owners who have to carefully respect the indications for themselves and for their companion animals (Marston et al., 2016).

## Antimicrobial resistance in new pets

Animals such as reptiles (turtles, snakes), rodents (hamsters, mice, guinea pigs), ornamental fish, indoor birds, rabbits and amphibians are nowadays kept as companion animals (Gwenzi et al., 2021). When adopting an exotic animal, account must be taken of its special needs in terms of environment, adequate food and freedom to express its behavior, as poor management of the pet can contribute to the development of stress and diseases, that are sometimes treated with inappropriate antibiotics as shown by data (Lim and Xie, 2021). Also the trade of these animals between countries has to be carried out following all the rules of healthcare, biosecurity and wellness in order to limit the spread of AMR (Bush et al., 2014).

Since they have only recently been introduced as pets (Lim and Xie, 2021), there is a great lack of information about these species (Gwenzi et al., 2021). Furthermore, it is possible to identify a small number of registered antimicrobial agents for these animals and the evidence-based medicine guidelines used to treat their diseases are also to be implemented (Damborg et al., 2016). In fact nowadays veterinarians often have to choose medical therapy based on their clinical experience on other animal species(Lim and Xie, 2021). What is worrying is that these new pets can transmit antimicrobial-resistant bacteria just like more common pets, such as dogs and cats, and food-producing animals (Chen et al., 2019; Gwenzi et al., 2021). The effective surveillance system for all antibiotics is a necessary measure that has to be introduced, in fact through a complete database including both antimicrobial agents registered for exotic species and those used off-label, it would be possible to have a better perception of the real condition of AMR in exotic animals, in order to adopt ASPs also for these species (Chen et al., 2019, Damborg et al., 2016). Exotic animals consist of different species, and few drugs are registered for them, so veterinarians have to choose off-label medicines or to require galenic preparations to allow the administration of extremely small quantities (Damborg et al., 2016; Papich, 2021). A similar problem is found in the potentially toxic effects to be avoided of some drugs that administered orally can lead to dysbiosis in some more sensitive species like rabbits, rodents and guinea pigs (Huynh et al., 2014; Papich, 2021). Because of all these difficulties, despite drugs such as fluoroquinolones and macrolides belonging to HPCIAs, are the most frequently prescribed and administered antibiotics in exotic animals (WHO, 2019).

Nowadays clinical practice of new pets needs to be enriched with all the most appropriate measures to allow proper management of these species while respecting their wellness and keeping in mind that they increasingly share the same environment with humans (Lim and Xie, 2020). Among the measures of ASPs of exotic animals (Lloyd and Page, 2018), alternative therapies are poorly experienced and could offer new effective strategies to limit the spread of antimicrobial resistance (Marston et al., 2016). Also, in this case, it is essential not to neglect the role of exotic animals as pets as a source and vehicle of AMR to other animals or to humans and they can be interpreted as sentinel to surveil AMR (Chen et al., 2019; Gwenzi et al., 2021).

## Wild animals

The existence of resistant bacteria is derived from natural resistance present in several bacteria, which has existed for millions of years, and it is an evolutionary consequence of bacterial competition with other microorganisms in their ecological niches (D'Costa et al., 2011; Torres et al., 2020). The impact of humans pushed the acquisition of resistance also bacteria normally present in wildlife: demographic changes associated with urbanization and poor sanitation, discharge of antibiotic residues through environmental wasting, and biocide use in livestock production contribute to this phenomenon (Marshall and Levy, 2011; Woolhouse et al., 2015). According to the global One Health approach, some authors already underlined the link between the emergence of AMR in humans and livestock and the AMR in wildlife (Jones et al., 2008) and others reported that wildlife species could represent a reservoir for resistant microorganisms and resistance genes (Vittecoq et al., 2016). Theoretically, wild animals are not treated with antibiotics but their association with humans, food producing animals, domestic animals directly or indirectly through humanized or urbanized environments, can enhance the passage of resistant commensal and pathogen bacteria (Torres et al., 2020). Moreover, some wild species are hunted and consumed and could be responsible for foodborne pathogens in humans due to manipulation and consumption of not well processed raw meat (Dias et al., 2015). A recent review underlined the increasing interest in searching for new correlations between wild animals and AMR, being focused on the fundamental role of the environment: wild species populations census and identification of high-risk areas will be the next steps to improve AMR surveillance (Torres et al., 2020). Wild animals could also be intended as sentinels of AMR in the environment. Recently, testudines have been considered to monitor the dissemination of AMR in marine water (Drane et al., 2021). The migratory nature of sea turtles permits them to exceed thousands of kilometers every year across several geographical areas (Witherington et al., 2009; Brasg et al., 2017). Wastewater derived from industry and agriculture is the major contributor to sea-water pollution: Spain, France, Italy, Greece, Croatia and Slovenia have implemented their One Health action plans in order to treat the wastewater prior the discharge into the Mediterranean Sea. The North African countries bordering the Mediterranean Sea are much less stringent in their regulation about treatment of water and about the use of antibiotics in agriculture thus leading to a possible contamination of the entire sea and increasing the risk to propagate antibiotic resistance genes (Foti et al., 2009). According to the aforementioned factors, it would be hard to establish a reliable assessment of the geographical origin of antimicrobial resistance (Drane et al., 2021). About the direct use of antimicrobial drugs in sea turtles, several of them are rescued and cured in rehabilitation centers that use broad spectrum antibiotic drugs, and rapid sensitivity tests specifically labeled for wild animals are still missing. Antibiotics belonging to quinolones and fluoroquinolones, beta lactams and tetracycline classes are often used and encompass the highest rate of resistance (Drane et al., 2021). These drugs are also frequently used in reptiles because of their safety profile but this led to another topic: limited information is available about pharmacokinetic and pharmacodynamic of antimicrobial drugs in wild or exotic animals, constraining veterinarians to routinely apply a few known therapeutic protocols. Recently, some authors investigated the pharmacokinetic-pharmacodynamic profiles of marbofloxacin and enrofloxacin in turtles (*Trachemis scripta scripta*) and in bearded dragons (*Pogona vitticeps*), respectively (Salvadori et al., 2016, Vercelli et al., 2016). It is interesting to note that even if these two fluoroquinolones expressed a very safe profile in these patients, the counterpart is very worrying: the cloacal swabs collected during the experiments were used to isolate and identify bacteria and to delineate the antimicrobial resistance pattern. The majority of commensal bacteria died after 24 hours from the administration of the drugs, leaving only pathogen and resistant *E.coli* and *Salmonella*.

## Environmental impact of AMR

A common way to transfer AMR among humans and animals is through the environment. This was well established but its role is still underestimated. Antibiotics and antibiotic-resistant pathogens are released into the environment through abandoned animals (i.e., cattle in India), stray animals and waste derived from livestock and agriculture (Kumar et al., 2021). The manure of treated animals could be used as fertilizer on the fields and can run into water, thus this can represent contamination for the human food chain (Hughes et al., 2013, Wellington et al., 2013). It was clearly established that antibiotic resistant bacteria can be present in water and soil, but further evaluations are needed to understand the impact on public health: for examples, it is necessary to comprehend if the presence of resistant bacteria in feces can be responsible of horizontal gene transfer to pathogens and to quantify the amount of AMR bacteria that can be disseminated through the environment (Kumar et al., 2021). People encounter resistant bacteria drinking contaminated water, consuming contaminated vegetables, fish and meat, or commensal flora can transfer plasmids or transposons encoding for resistance to pathogens present in the bowel of the host (Wellington et al., 2013). This last condition has been already described for commensal bacteria and pathogens that can share macrolide-resistance genes *ermB, ermF* and *ermG* and the tetracycline-resistance genes *tetM* and *tetQ* (Kumar et al., 2021). However, the exact mechanism of the transfer of resistance genes in the gut is still poorly understood. The passage of resistant genes has been proven between *Bacteroides* and pathogenic *E. coli* strain only in i*n vitro* laboratory conditions and failed in *in vivo* experiments (Kumar et al., 2021).

The ecological nature of antimicrobial resistance is a reflection and a consequence of interplay of different forms of life on the planet: some resistance mechanisms such as beta lactamases, are million years old (Perry and Wright, 2014). Even if the presence of resistant mechanisms could be dated prior to the antibiotic era, it could not be denied that human activity had and still has an impact to select the resistome, which is the totality of resistant genes in the wider environment (Ruuskanen et al., 2016; Wellington et al., 2013). Pollutants, such as heavy metals, quaternary ammonium compounds, antifouling agents, and detergents might affect the frequencies of antibiotic resistance through lined selection, even at low concentrations (Pieri et al., 2020). In high income countries people have limited options of direct contact with food producing animals, since the transmission of resistance has a foodborne origin from agricultural sources with contamination on the field (considering vegetables and fruits) or contamination of meat (beef, swine and poultry) at the slaughterhouse (Nelson et al., 2007). In developing countries, drinking water represents the main source of transmission of resistant bacteria or genes for animals and humans (Finley et al., 2013). Poor sanitation and poor hygiene procedures can allow direct transmission from person to person: an important and underestimated source are travelers that return home colonized with bacteria acquired abroad (Collignon and Kennedy, 2015). This concept could be easily transposed to animals that in globalized trade could be transported worldwide or for wild animals that can run across long distances in migratory routes (Collignon and McEwen, 2019).

## Current issues and new strategies in the post antibiotic era

Considering the literature and the aforementioned factors, it seems clear that the problem of antibiotic resistance represents a global concern for public health and that veterinary medicine is involved and should be more and more involved in the future to find new strategies in the “Post antibiotic era”.

Despite the recognition of the problem, there is still the need to standardize microbiology methods in veterinary medicine. Among the different issues that arose in the last decades, the rational use of antibiotic drugs represents a milestone, but to use these drugs in a rational way, it is necessary to have good diagnostic tools that can orient the choice of the most specific and targeted therapy. This point is clear, and it is the focus of the major ASPs that were born in the last years. The recent paper of Timofte and colleagues highlighted the necessity to standardize the methodologies and the collection techniques of different specimens in the veterinary laboratories in order to have harmonized results about antimicrobial susceptibility testing (AST), to have specific guidelines, to identify the mechanisms of resistance typical of veterinary pathogens, and to carefully train specialists in veterinary microbiology (Timofte et al., 2021). The necessity to involve specialists in the fight against antibiotic resistance emerged also in the review of Lees and colleagues focusing on veterinary pharmacology (Lees, Pelligand, Giraud, & Toutain, 2019). It has been reported that the importance to create an antibiotic stewardship team is fundamental to carefully study the specific situation, not only related to the species (i.e., small or large animals, food producing animals, exotic or wild animals) but also to the pathology, the etiological agent and the geographic area (Guardabassi and Prescott, 2015; Vercelli et al., 2021). A reliable workflow, able to guide clinicians in sample collection and interpretation of laboratory tests is still lacking. Two recent papers demonstrated that the introduction of the online antimicrobial stewardship program, which gives advice and recommendations, significantly decreased the prescription of antimicrobials for dogs and cats (Hubbuc et al., 2020; Lehner et al., 2020).

The interpretation of surveillance data is still challenging due to the lack of an harmonized system among veterinary microbiology laboratories, and it is particularly evident trying to compare the results obtained from AST following the guidelines of European Committee on Antimicrobial Susceptibility Testing (EUCAST) with others following the American Clinical and Laboratory Standards Institute (CLSI) (Compri et al., 2020). The use of multiple standards is a limitation not only between laboratories but also among countries, misleading the clinicians in the optimal therapeutic choice and compromising the global surveillance of AMR (Timofte et al., 2021). Moreover, specific clinical breakpoints related to veterinary pathogens and species are still missing even if the importance of these topics have been recognized and subcommittees of CLSI and EUCAST (VAST and VETCAST, respectively) have been created to achieve this goal (Timofte et al., 2021). Ideally, laboratory procedures, guidelines and interpretation of the results should be standardized at European level similarly to the human system Microbiology Investigation Criteria for Reporting Objectively (MICRO) (Turner et al., 2019). Several points raised in this paragraph have been taken into consideration in the action plan of the European Network for Optimization of Veterinary Antimicrobial Treatment (ENOVAT) that is a common plan that brings together veterinary specialists in microbiology, pharmacology, and epidemiology to build new antimicrobial stewardship programs (https://enovat.eu/).

Another issue could be to better establish guidelines for laboratory testing, such as minimal inhibitory concentration (MIC). MIC provides an important tool for surveillance of phenotypic resistance, allowing for assessment of trends on antimicrobial resistance phenomenon. MIC will give important insight about the shift of antimicrobial resistance, also related to the clinical and surveillance settings (Michael et al., 2020). In order to potentiate this tool, several methodologies could be applied: complex models can be mixed together for surveillance programs while more simple models such as logistic regression can allow to integrate data from different sources and to compare prevalence of MICs classified as resistant phenotypes in order to give immediate clinical classifications. This led to the fact that clinical breakpoints can be updated to understand if a treatment can achieve a therapeutic outcome (Michael et al., 2020).

Several studies have emphasized the importance of mutant prevention concentration (MPC)-based dosing approach to improve therapeutic outcome and limit the selection of resistant mutant bacteria (Awji et al., 2012). This concept is correlated to the mutant selection window (MSW) that describes how drug exposures below the MPC may induce the selection of resistant bacterial strains. This hypothesis is based on the fact that drug-resistant mutant subpopulations, present before the initiation of antimicrobial treatment, are enriched and amplified during therapy when antimicrobial concentrations fall within the specific range of MSW. The upper boundary of the MSW is the MIC of the least drug-susceptible mutant subpopulation, and the lower boundary of the MSW is the lowest concentration that blocks the growth of the majority of drug-susceptible bacteria, often approximated by the minimal concentration that inhibits colony formation by 99% (MIC_99_; Awji et al., 2012).

**Fig. 2:**
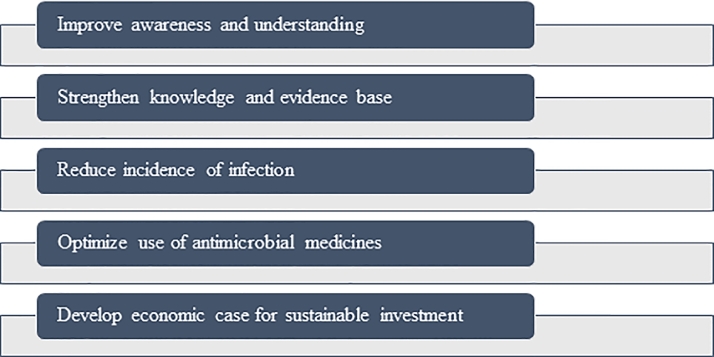
The figure represents the five key points of the educational plans for professionals and consumers designed by World Health Organization (WHO) global action plan (adapted by Collignon and McEwen, 2019)

At global level, the WHO and the World Organization for Animal Health have designed several protocols to optimize antimicrobial use, to give advice to national governments and to improve surveillance programs (World Health Organization 2021). The main message is to reduce dramatically the use of antimicrobial drugs because it is presupposed that the antimicrobial drug consumption is the primary driver of the emergence and the widespread of AMR: reducing the use, the AMR phenomenon would proportionally decrease (Noyes et al., 2021). The situation is much more complex because the strict relationship between antimicrobial consumption and AMR is misleading. The One Health approach summarizes this complexity and the only way to find a solution is pursuing judicious collaboration among several disciplines, frameworks, and regulatory systems. Thus, the reduction of consumption is necessary but should be strictly correlated to the clinical outcomes and each therapy should be tailored for specific patients, pathology, and geographic areas (Noyes et al., 2021). A narrow intervention encoded careful, stepwise, and continuous monitoring that unfortunately does not produce shortcoming effects (Noyes et al., 2021). The necessity to expand the research is clear considering that interhost AMR transmission between anthropogenic source and animal population is the predominant driver of AMR in many situations (Collignon and Beggs, 2019). This led to open a discussion to implement public health measures, to have better access to clean water, better housing, less crowding, safer foods, less transmission in hospitals by adopting better infection control and prevention practices, more detailed regulations, communications with citizens that can be involved as patients or as consumers (Collignon and Beggs, 2019) and a better training of professionals, especially for veterinary students (Espinosa-Gongora et al., 2021). All the aforementioned factors are summarized in five key points by WHO action plan (Fig. 2).

The critical issues related to the alternative strategies to fight antimicrobial resistance are related to reducing the passage of resistance or genes of resistance between animals and humans. It has been widely investigated the possibility to use bacteriophages, antimicrobial peptides or bacteriocins, antimicrobial adjuvants, fecal microbiota transplant and competitive exclusion of pathogen using pre - and probiotics (Kumar et al., 2021). Another perspective is represented by a bacterial secretion system that is a highly specialized nano-mechanical system that is capable of direct delivery of substances in eukaryotic cells (Kumar et al., 2021).

In farm animals, phage therapy efficacy perspectives have been widely studied specifically to control the spread of zoonoses, to treat diseases and to limit economic losses while in pets a few studies have been conducted (Loponte et al., 2021).

Specifically related to the food producing animals, antibiotic usage can be reduced without reducing productivity and profitability: prudent use, complementary strategies to increase animal welfare, hygiene practice, administration of probiotics and vaccines can significantly reduce the usage of antibiotics (Levy, 2014).

## Conclusion

The present review aimed to summarize the main topics related to antimicrobial resistance, underlying all the aspects in which the role of veterinarians is fundamental. The solution to AMR is far to be found and it is not unique. One Health approach is mandatory to include the plethora of factors and to give them the right importance. In the past, the use of antibiotics in livestock has been considered the only responsible for the widespread of AMR but also pets, environment, wild animals and exotic animals has a key role, often underestimated but not negligible.

The role of veterinary specialists, such as microbiologists, pharmacologists and epidemiologists, has to be improved and better emphasized. Educational tools have to be carefully applied not only among professionals but also for consumers and citizens.

## Funding

This research did not receive any specific grant from funding agencies in the public, commercial, or not-for-profit sectors.

## Conflict of interest

The authors declare no conflict of interest.

## Declaration of Competing interests

The authors declare that they have no known competing financial interests or personal relationships that could have appeared to influence the work reported in this paper.
